# Systematic Comparison of Molecular Conformations of H^+^,K^+^-ATPase Reveals an Important Contribution of the A-M2 Linker for the Luminal Gating*

**DOI:** 10.1074/jbc.M114.584623

**Published:** 2014-09-17

**Authors:** Kazuhiro Abe, Kazutoshi Tani, Yoshinori Fujiyoshi

**Affiliations:** From the ‡ Cellular and Structural Physiology Institute and; § Graduate School of Pharmaceutical Science, Nagoya University, Nagoya 464-8601, Japan

**Keywords:** ATPase, Crystal Structure, Electron Microscopy (EM), Membrane Protein, Protein Structure, H+,K+-ATPase, P-type ATPase, Electron Crystallography, Gastric

## Abstract

Gastric H^+^,K^+^-ATPase, an ATP-driven proton pump responsible for gastric acidification, is a molecular target for anti-ulcer drugs. Here we show its cryo-electron microscopy (EM) structure in an *E2*P analog state, bound to magnesium fluoride (MgF), and its K^+^-competitive antagonist SCH28080, determined at 7 Å resolution by electron crystallography of two-dimensional crystals. Systematic comparison with other E*2P*-related cryo-EM structures revealed that the molecular conformation in the (SCH)*E2*·MgF state is remarkably distinguishable. Although the azimuthal position of the A domain of the (SCH)*E2*·MgF state is similar to that in the *E2*·AlF (aluminum fluoride) state, in which the transmembrane luminal gate is closed, the arrangement of transmembrane helices in the (SCH)*E2*·MgF state shows a luminal-open conformation imposed on by bound SCH28080 at its luminal cavity, based on observations of the structure in the SCH28080-bound *E2*·BeF (beryllium fluoride) state. The molecular conformation of the (SCH)*E2*·MgF state thus represents a mixed overall structure in which its cytoplasmic and luminal half appear to be independently modulated by a phosphate analog and an antagonist bound to the respective parts of the enzyme. Comparison of the molecular conformations revealed that the linker region connecting the A domain and the transmembrane helix 2 (A-M2 linker) mediates the regulation of luminal gating. The mechanistic rationale underlying luminal gating observed in H^+^,K^+^-ATPase is consistent with that observed in sarcoplasmic reticulum Ca^2+^-ATPase and other P-type ATPases and is most likely conserved for the P-type ATPase family in general.

## Teaser

**Background:** The gastric H^+^,K^+^-ATPase proton pump achieves gastric acid secretion.

**Results:** A newly determined (SCH)*E2*·MgF structure represents a hybrid conformation of (SCH)*E2*·BeF and luminal-closed (Rb^+^)*E2*·AlF states.

**Conclusion:** Comparison of *E2*P-related structures revealed that the A-M2 linker importantly contributes to conformational changes of the enzyme.

**Significance:** A-M2 linker-mediated conformational change is conserved between H^+^,K^+^-ATPase and Ca^2+^-ATPase and perhaps other P-type ATPases.

## Introduction

**FIGURE 1.**
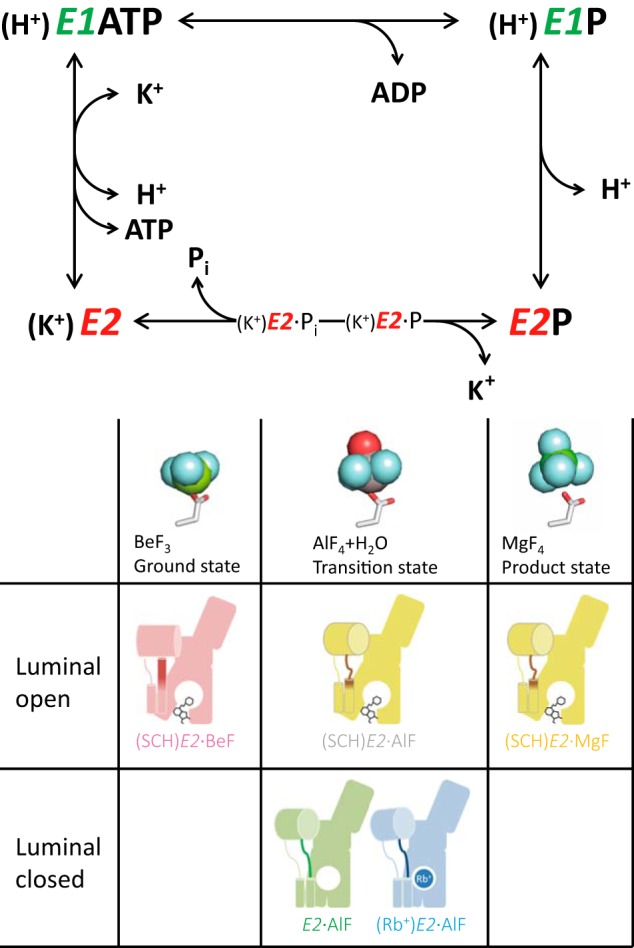
**Transport cycle of gastric H^+^,K^+^-ATPase and reaction sub-steps induced by XFs.** *Upper panel*, ion transport and ATP hydrolysis are coupled to the cyclic conformational conversion of the enzyme (abbreviated as *E*) between its main states, *E1* and *E2*, and their respective auto-phosphorylated forms, *E1*P and *E2*P. ATP hydrolysis generates the phosphoenzyme intermediate (*E1*P, *E2*P) by transfer of the γ-phosphate to the invariant Asp-386 residue in the presence of Mg^2+^ (omitted in the figure for simplicity). *Lower panel*, *E2*P and its dephosphorylation steps mimicked by XFs are shown (see “*Cytoplasmic Domains*” for details). The atomic models represent the coordination chemistry of XFs (*sticks* for an aspartate residue and *spheres* for BeF_3_ (*left*), AlF_4_ and a water (*middle*), and MgF_4_ (*right*), respectively). Schematics of the molecular conformations presented in this study are shown (see Fig. 9 for details).

Gastric H^+^,K^+^-ATPase is an ATP-driven proton pump responsible for the gastric acid secretion (1, 2). This enzyme catalyzes the energetically uphill, electro-neutral exchange of H^+^/K^+^ coupled with ATP hydrolysis, which generates a million-fold H^+^-gradient across the parietal cell membrane (*i.e.* pH 1 in the stomach *versus* pH 7 in the cytosol; see Refs. 3 and 4). Like other P-type ATPases, the enzyme undergoes cyclical conformational changes between two principal reaction states (*E1*, *E2*) and their auto-phosphorylated forms (*E1*P, *E2*P) during its transport cycle (see Fig. 1 and Refs. 5–7).

H^+^,K^+^-ATPase comprises both an α- and an β-subunit. Like other P2-type ATPases (8), including the housekeeping sodium pump (Na^+^,K^+^-ATPase; Ref. 9) and sarcoplasmic reticulum Ca^2+^-ATPase 1a (SERCA^2^; Ref. 10), the catalytic α-subunit contains 10 transmembrane (TM) helices in which cation binding sites are located, and three cytoplasmic domains (A, P, and N domains) where ATP hydrolysis and the accompanying autophosphorylation occur (11). In addition to the α-subunit, H^+^,K^+^-ATPase and Na^+^,K^+^-ATPase require an accessory β-subunit that is indispensable for functional expression and trafficking of the αβ-complex to the cell surface (12). Furthermore, recent studies of H^+^,K^+^-ATPase revealed important contributions of the β-subunit for regulating the *E1*P/*E2*P equilibrium, which is an important characteristic for generating the large H^+^ gradient between gastric membranes (13, 14).

Because the first atomic model of SERCA was reported in 2000 (15), studies of its structure and function have revealed its detailed molecular mechanisms, with crystal structures covering almost the entire reaction cycle (16). Several cryo-EM structures of H^+^,K^+^-ATPase have been determined by electron crystallography of two-dimensional crystals, allowing for evaluation of its important physiologic roles (13, 17–19). The conformational changes of H^+^,K^+^-ATPase that accompany its transport cycle, however, are not yet fully elucidated.

To clarify the transport mechanism of H^+^,K^+^-ATPase, a better understanding of the *E2*P state structure as a key reaction intermediate is essential to reveal the conformational changes that occur for the H^+^/K^+^ exchange against a luminal solution with opening and closing of the transport pathway and gate. Extensive studies of SERCA with fluorinated phosphate analogs (XFs) led to the characterization of its structural and functional properties in various states (Fig. 1 and Refs. 20–22). Tetrahedral BeF_3_ (BeF) shows the closest coordination of aspartyl phosphate (*E2*·BeF, *E2*P ground state), a trigonal bipyramidal complex of AlF_4_ and water (AlF), which is assigned as a transition analog from aspartyl phosphate to its hydrolysate (*E2*·AlF, *E2*·P transition state), and a tetrahedral MgF_4_ (MgF), which mimics the inorganic phosphate produced by *E2*P hydrolysis (*E2*·MgF, *E2*·P_i_ product state). Because each bound XF is tightly coordinated by a TGES loop located at the A domain, subtle differences in their environment generate a different type of cytoplasmic arrangement, leading to distinct conformational changes of the whole enzyme structure (16, 21). Therefore, even at a limited resolution of ∼8 Å determined by electron crystallography, molecular events occurring at the phosphorylation site and the luminal gate can be observed based on the rearrangement of the cytoplasmic domains and TM helices, respectively, in response to each bound XF (13, 17–19).

Here we determined new cryo-EM structures of H^+^,K^+^-ATPase using a phosphate analog (either AlF or MgF) and a specific antagonist, SCH28080. Although these structures represent an identical conformation, their cytoplasmic and luminal halves appeared to be modulated independently by the phosphate analog and the bound antagonist. Systematic comparison of the cryo-EM structures in the *E2*P-related states allows us to propose a luminal gating mechanism of H^+^,K^+^-ATPase.

## EXPERIMENTAL PROCEDURES

### 

#### 

##### Isolation of H^+^,K^+^-ATPase-enriched Membrane Fractions

Purification of the H^+^,K^+^-ATPase-enriched membrane fractions (G_1_ and G_2_) was performed as described previously (23) with some modifications, and the fractions were further purified with SDS (24). Purified membrane fractions were frozen in liquid nitrogen and stored at −80 °C until use. For two-dimensional crystallization and other biochemical analyses, an SDS-purified G_1_ fraction was used as described previously (13).

##### Two-dimensional Crystallization and Image Analysis

An SDS-purified G_1_ fraction (8 mg/ml of protein) was solubilized for 10 min on ice with 8 mg/ml octaethylene glycol dodecyl ether (C_12_E_8_, Nikko Chemical, Japan) in 40 mm MES, 20 mm Mg(CH_3_COO)_2_, 5 mm ATP, 10% (v/v) glycerol, and 3 mm dithiothreitol (DTT) at pH 5.5 adjusted by Tris. After removing the insoluble material by ultracentrifugation at 186,000 × *g* for 20 min, the supernatant was mixed with dioleoylphosphatidylcholine (Avanti) at a lipid-to-protein ratio (w/w) of 1.0–1.2. The samples were then placed in 10-μl microdialysis buttons (Hampton Research) using a dialysis membrane with a molecular mass cutoff of 25 kDa (SPECTRA/Pro #7, SPECTRUM) and first dialyzed against 10 mm MES, 10% (v/v) glycerol, 1 mm MgCl_2_, 1 mm AlCl_3_, 4 mm NaF, 1 mm ADP, 3 mm DTT, and 1 μm SCH28080 at pH 5.5 adjusted by Tris on ice for 1 day and then against 20 mm propionate, 1 mm MgCl_2_, 1 mm AlCl_3_, 4 mm NaF, 1 mm ADP, 3 mm DTT, and 10 μm SCH28080 at pH 4.8 with Tris at 3 °C for 12–16 days for two-dimensional crystallization of the (SCH)*E2*·AlF state. For two-dimensional crystallization of the (SCH)*E2*·MgF state, 1 mm MgCl_2_, 1 mm AlCl_3_, and 4 mm NaF were replaced with 5 mm MgCl_2_ and 10 mm NaF.

Samples were negatively stained with 2% (w/v) uranyl acetate to screen for crystallization conditions. Specimens for cryo-EM were prepared in a cold room using the carbon sandwich method (25, 26). Images were recorded with a JEM-3000SFF electron microscope (JEOL) equipped with a super fluid helium stage (27, 28) on SO-163 film (Carestream Health). Digitized images were processed with MRC image processing programs (29), and their initial contrast transfer function parameters were determined (30) for correction. The data tilted to 60° were merged using LATLINE (31) and used to calculate a three-dimensional density map at 8 Å and 7 Å resolution for the (SCH)*E2*·AlF and (SCH)*E2*·MgF states, respectively (Table 1). The EM density maps of (SCH)*E2*·AlF and (SCH)*E2*·MgF were deposited in the EMDataBank (accession code EMD-2759 and EMD-2760, respectively). The coordinates of the homology models were also deposited in the PDB (accession code 4UX1 and 4UX2, respectively).

##### Homology Models and Structural Comparison of E2P-related States of H^+^,K^+^-ATPase

Homology models of (SCH)*E2*·AlF and (SCH)*E2*·MgF were built with MODELLER v9.12 (32) using the atomic model of Na^+^,K^+^-ATPase with K^+^ and ouabain (Ref. 33, PDB code 3A3Y) as a starting template. The initial manual fitting of the homology model into the EM density map was achieved using COOT (34). The adjustment for each individual domain and TM helix with the EM density map was performed using SITUS (35). After the positional search, further fine-fitting and connecting of the split loop region were performed manually using COOT with regularization refinement. For systematic comparison with other cryo-EM structures, the same procedures were applied to build homology models for the (SCH)*E2*·BeF state and (Rb^+^)*E2*·AlF states using atomic models of Na^+^K^+^-ATPase in the high affinity ouabain binding state (Ref. 36, PBD code 4HYT) and K^+^-bound *E2*·MgF state (Ref. 37, PDB code 2ZXE), respectively, although the resulting models are almost identical to those reported previously (18, 19).

To compare the relative orientation of the cytoplasmic domains, the EM density map and homology models were aligned based on the superpositioning of the molecules on their P domain (see Fig. 3). To compare other structural parts, including the A-M2 linker and TM helices, the molecules were aligned by superpositioning of the TM segments M7-M10 and βM, which are the least variable of the reported SERCA structures.

##### Fluorescein 5′-Isothiocyanate (FITC) Fluorescence Measurement

FITC modification of H^+^,K^+^-ATPase was performed as follows (38–40). Purified membrane fractions containing H^+^,K^+^-ATPase (0.5 mg/ml) were incubated in 1 mm EDTA, 100 mm Tris/HCl (pH 9.2), 0.25 m sucrose, and 10 μm FITC dissolved in Me_2_SO at 25 °C for 30 min. The modification was terminated by the addition of 1 mm β-mercaptoethanol, and the samples were washed twice with 10 mm HEPES/Tris (pH 7.0), 1 mm EDTA, and 0.25 m sucrose.

The change in FITC fluorescence at the steady state was determined at 37 °C in a 3-ml solution containing 50 mm HEPES/Tris, pH 7.0, 1 mm MgCl_2_, 25 mm sucrose, and 20 μg of the FITC-labeled H^+^,K^+^-ATPase membrane fraction. Reactions were initiated by adding 5 mm MgCl_2_ and 5 mm P_i_ for “Mg^2+^ + P_i_ (*E2*P),” 1 mm BeSO_4_ and 4 mm NaF for “BeF (*E2*·BeF),” 1 mm AlCl_3_ and 4 mm NaF for “AlF (*E2*·AlF),” or 5 mm MgCl_2_ and 10 mm NaF for “MgF (*E2*·MgF)” conditions followed by 10 μm SCH28080. The addition of the reagents had a negligible effect on the fluorescence intensity of the FITC dye itself. All FITC fluorescence measurements were performed using a Shimadzu F5500 spectrophotometer fitted with a magnetic device to stir the cuvette contents and control the water temperature. The FITC fluorescence intensity was optimized by measurement at the wavelength pair (excitation_495 nm_/emission_515 nm_) with the slit width set to 5 nm for either excitation or emission.

##### Enzyme Activity

H^+^,K^+^-ATPase activity and K^+^-*p*-nitrophenyl phosphatase activity of membrane-bound enzyme were determined as described previously (17, 39). For the measurement of the dose-dependent inhibition by XFs, FITC-modified membrane preparations were incubated in the indicated concentrations of BeSO_4_ or AlCl_3_ in the presence of 1 mm NaF, 0.1 mm MgCl_2_, 250 mm sucrose, and 40 mm HEPES/Tris, pH 7.0. For MgF, an equal amount of MgCl_2_ and NaF was used. After incubation for 1 h at 37 °C, residual K^+^-*p*-nitrophenyl phosphatase activity was measured colorimetrically (20 mm *p*-nitrophenyl phosphate/Tris, 20 mm MgCl_2_, 16 mm KCl, 250 mm sucrose, and 40 mm imidazole/HCl, pH 7.8).

##### Measurement of [^3^H]SCH28080 Binding

In equilibrium binding experiments, to determine the *K_d_* value (41, 42), the purified membrane fractions (5–100 μg/ml) were suspended in a buffer comprising 40 mm MES (pH 6.5, adjusted with Tris), 2% glycerol, and the addition of 5 mm MgCl_2_ and 5 mm P_i_ for “MgP_i_,” 1 mm MgCl_2_, 1 mm BeSO_4_, and 4 mm NaF for “BeF,”; 1 mm MgCl_2_, 1 mm AlCl_3_, and 4 mm NaF for “AlF,” or 5 mm MgCl_2_ and 10 mm NaF for “MgF” followed by a 30-min incubation at room temperature. Each reaction tube was then incubated at 0–37 °C, and 5 nm--1 μm [^3^H]SCH28080 (synthesized by PerkinElmer Life Sciences) was added. The level of nonspecific binding was determined in the presence of a 100-fold excess of unlabeled SCH28080 over the concentration range of [^3^H]SCH28080 used. The enzyme suspension (0.1–2 ml) was incubated for 1 h at the indicated temperature and rapidly filtered through a nitrocellulose membrane filter (HAWP Millipore filter, 0.45 μm) pre-wetted with washing buffer comprising 10 mm MES (pH 6.5, adjusted with Tris), 1 mm MgCl_2_, and 10% PEG 3500, which was placed on top of a glass fiber filter. The membrane was washed 3 times with 5 ml of washing buffer to remove unbound inhibitor. The membrane was placed in a 20-ml scintillation vial; 1 ml of 2% SDS was added to dissolve the H^+^,K^+^-ATPase and bound SCH28080 from the filter membrane, and 10 ml of scintillation solvent was added and the contents counted. Binding of [^3^H]SCH28080 was assessed by subtracting the level of nonspecific binding of [^3^H]SCH28080, obtained in the presence of a 100-fold excess of nonradioactive SCH28080, from the amount of [^3^H]SCH28080 bound to the membrane in the absence of the cold inhibitor. The dose dependence of SCH28080 binding was fit to the hyperbolic binding curve, and the binding maximum (*B*_max_) and dissociation constant (*K_d_*) were determined at the indicated temperatures. The *B*_max_ values were 2.0–2.4 nmol/mg regardless of added XFs and reaction temperature, which was close to the maximum amount of *E*^32^P formed from [γ-^32^P]ATP (∼2 nmol/mg for the purified membrane fractions; Ref. 43) and thus consistent with previous results (42).

Overall changes in the enthalpy (Δ*H*) and entropy (Δ*S*) of SCH28080 were obtained from the van't Hoff plot using the relationship (41),

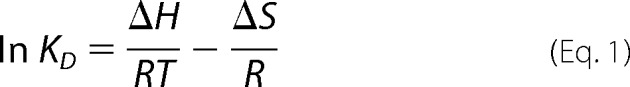
 where *R* is the gas constant.

Free energy terms were calculated using the equation,

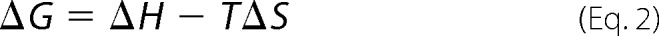


## RESULTS

### 

#### 

##### Two-dimensional Crystallization of (SCH)E2·AlF and (SCH)E2·MgF States

**FIGURE 2.**
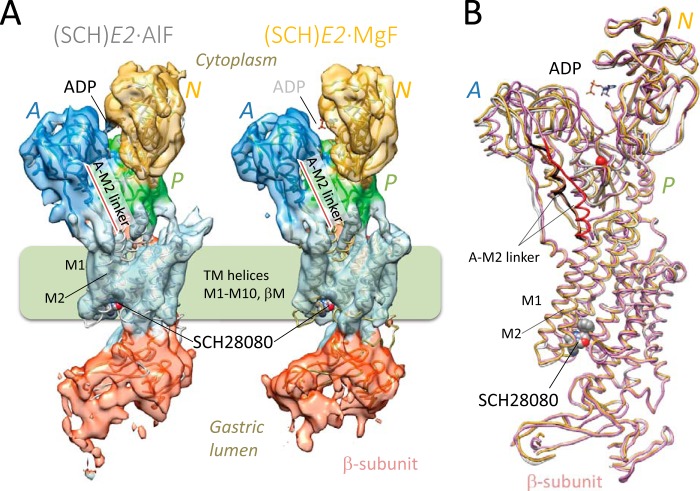
**Cryo-EM structure of H^+^,K^+^-ATPase in the (SCH)*E2*·AlF and (SCH)*E2*·MgF states.** *A*, overall structures of the H^+^,K^+^-ATPase in the (SCH)*E2*·AlF (*left*) and (SCH)*E2*·MgF (*right*) states analyzed at 8 Å and 7 Å resolution, respectively. The molecular surface represents the EM density map (contoured at 1 σ) with a superimposed homology model of H^+^,K^+^-ATPase (*ribbons*) in which individual domains and TM helices are fitted independently according to each EM density map (see “Experimental Procedures”). The A-M2 linker is highlighted by a *red bar*. The *light green box* indicates the approximate location of the lipid bilayer. *Blue*, A domain; *green*, P domain; *light blue*, TM helices; *red*, β-subunit. *B*, comparison of the homology models between the (SCH)*E2*·AlF (*gray*) and (SCH)*E2*·MgF (*yellow*) states reveals almost identical molecular conformations in either states, but these are significantly different from that of the (SCH)*E2*·BeF state (*pink*) regarding the location of the A domain and the conformation of the connecting A-M2 linker (highlighted by the darker color in each state). The autophosphorylation site (Asp-386) is indicated as a *red sphere*. In both panels, bound SCH28080 at the luminal cavity and ADP at the N domain are indicated as *spheres and stick representations*, respectively. The binding mode of SCH28080 is based on the previously reported docking simulation using a homology model of the (SCH)*E2*·BeF state (18).

Previously, we reported several cryo-EM structures of H^+^,K^+^-ATPase bound to different XFs in the presence or absence of ions and substrates for the TM domain (such as transported cation K^+^, its congener Rb^+^, and a specific antagonist SCH28080), which include the reaction state analog of *E2*·BeF (17), *E2*·AlF (13), (SCH)*E2*·BeF (18), and (Rb^+^)*E2*·AlF (19). For systematic comparison of the molecular conformations induced by XFs mimicking the reaction substeps of *E2*P dephosphorylation, we obtained two-dimensional crystals in the presence of SCH28080 in combination with either AlF or MgF and determined their three-dimensional structures at 8 Å and 7 Å resolution, respectively (Fig. 2*A*, Table 1). It is notable that no two-dimensional crystals were produced in the presence of either MgF alone or MgF with Rb^+^ (not shown). Because inhibition of H^+^,K^+^-ATPase activity by MgF is reversible and weaker than that of other XFs (17), stabilization of the molecular conformation by SCH28080 is required for the two-dimensional crystal formation when MgF is used as the phosphate analog. The two newly determined structures, however, were almost identical in their overall conformation, including the arrangement of the TM helices, the azimuthal position of the A domain, and the connecting linker between them (Fig. 2, *A* and *B*). In fact, x-ray structures of SERCA in the *E2*·AlF and *E2*·MgF states also indicated identical molecular conformations (21). Therefore, we concluded that these two structures of H^+^,K^+^-ATPase adopt an indistinguishable conformation and thus used the (SCH)*E2*·MgF structure as a representative with better crystallographic characteristics for subsequent comparison of the molecular conformation with other cryo-EM structures.

**TABLE 1:**
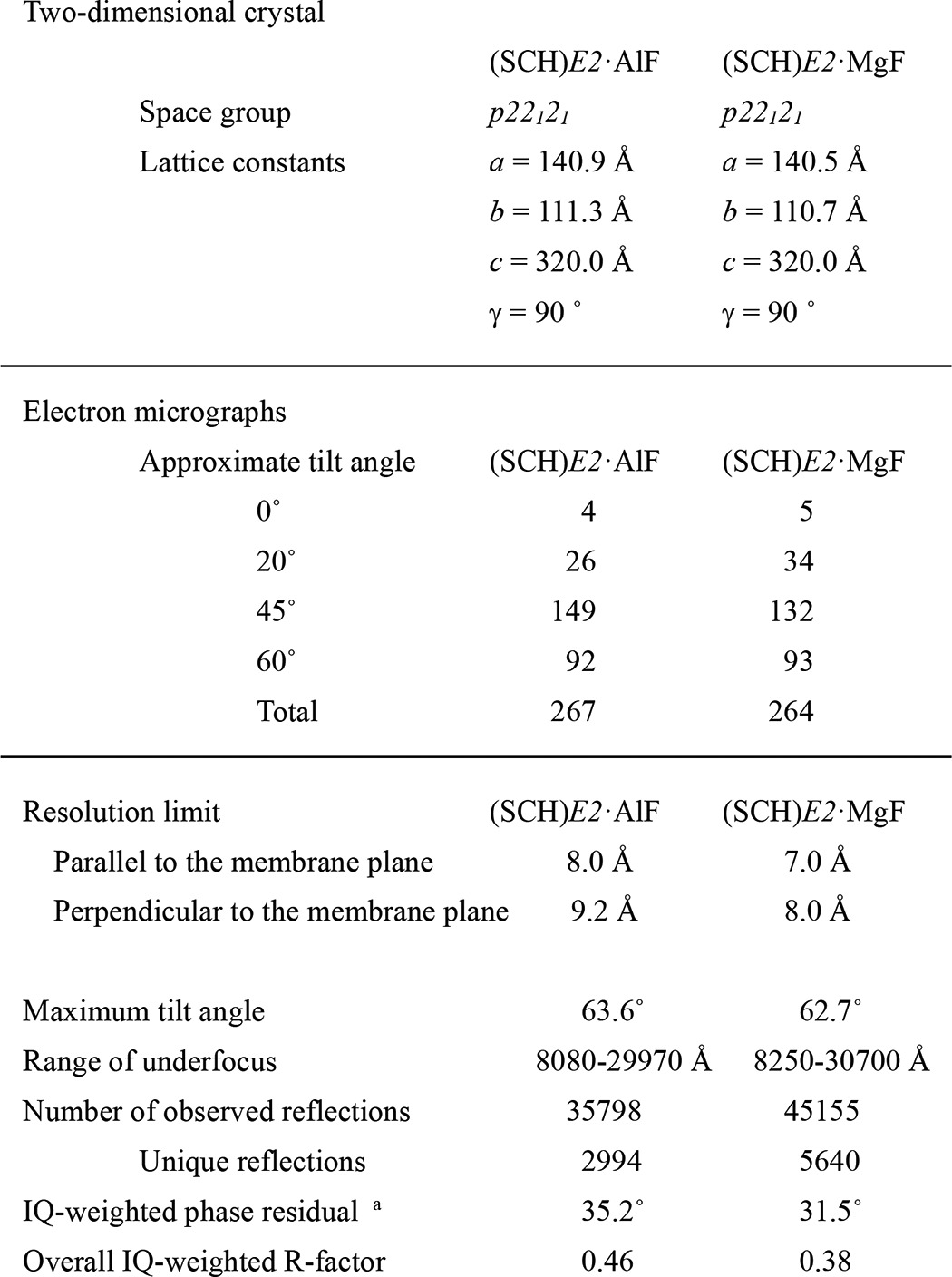


##### Cytoplasmic Domains

**FIGURE 3.**
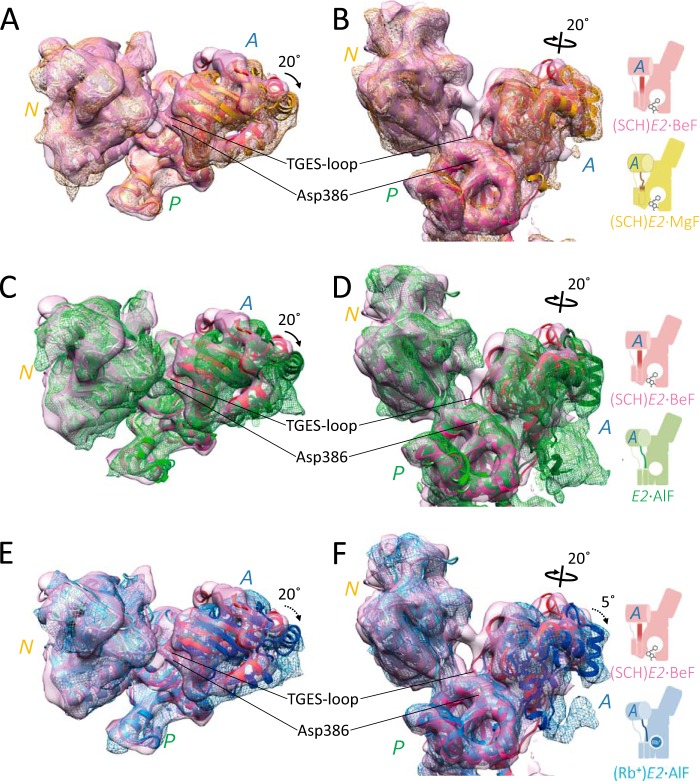
**Rotation of the A domain in response to bound XF.** Comparisons of the relative orientations of the cytoplasmic domains in the EM density maps of H^+^,K^+^-ATPase between the (SCH)*E2*·BeF (*pink surface*) *versus* (SCH)*E2*·MgF (*A* and *B*, *yellow mesh*), *E2*·AlF (*C* and *D*, *green mesh*), and (Rb^+^)*E2*·AlF (*E* and *F*, *blue mesh*) states superimposed with their respective homology models (*ribbons*, drawn in same color). *Black arrows* indicate the different (20°) azimuthal positions of the A domain between the (SCH)*E2*·BeF state *versus* other indicated states. The *dotted arrow* indicates a 5° inclination of the A domain during transition from the (SCH)*E2*·MgF state to the (Rb^+^)*E2*·AlF state. Molecules are aligned by superpositioning of the P domain and viewed from the cytoplasmic side in *A*, *C*, and *E* or from parallel to the membrane plane in (*B*, *D*, and *F*). Schematic representations of their conformational states are shown on the *right* (see Fig. 9 for details).

Like inorganic phosphate, XFs bind to the Asp residue in the invariant sequence of DKTG located at the P domain, and each bound XF is covered by the outermost TGES loop on the A domain. The different coordination chemistries of the XFs (Fig. 1) change the relative orientation of the XF-bound aspartate residue and TGES-loop, and thus the P and A domains assume different relative positions in respective XF-bound structures (21). When the *E2*P-related cryo-EM structures of H^+^,K^+^-ATPase were aligned according to their P domain, the location of the A domain was significantly different (Fig. 3, *A* and *B*, and Ref. 18). The A domain in (SCH)*E2*·MgF was rotated ∼20° around an axis approximately perpendicular to the membrane plane compared with that in the *E2*P ground state analog (SCH)*E2*·BeF (18) with its TM helices showing a luminal-open conformation. This azimuthal position of the A domain in (SCH)*E2*·MgF was similar to that in the luminal-closed *E2*·AlF (Fig. 3, *C* and *D*, and Ref. 13) and (Rb^+^)*E2*·AlF (Fig. 3, *E* and *F*, and Ref. 19) states, likely reflecting the difference in the coordination geometry between BeF and the other XFs (AlF and MgF). Furthermore, the A domain in (Rb^+^)*E2*·AlF was more inclined (∼5°) toward the membrane surface than that in (SCH)*E2*·MgF (Fig. 3*F*). This inclination of the A domain induced the TGES loop to segregate even further from the phosphorylation site, which may allow for water penetration at this site, facilitating the hydrolysis of the aspartyl phosphate. Hence, the observed inclination of the A domain induced by the Rb^+^ binding (19) in the H^+^,K^+^-ATPase (Rb^+^)*E2*·AlF state suggests that this conformation is a transient point that leads to the formation of the dephosphorylated (K^+^)*E2* state. Observed differences in the azimuthal positions of the A domain in *E2*P analog states of H^+^,K^+^-ATPase were consistent with reported SERCA structures (21, 22); thus a similar mechanism can be applied for the relationship between the phosphorylation states and the arrangement of the cytoplasmic domains in both ATPases.

##### Monitoring the Enzyme Conformation by the FITC Fluorescence Change

**FIGURE 4.**
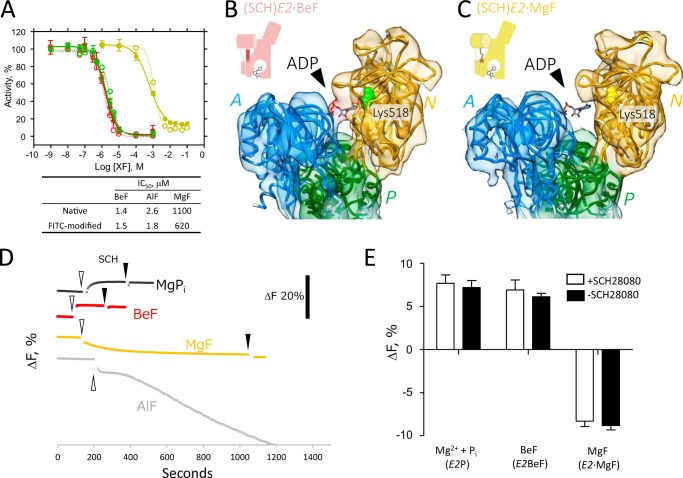
**Conformational change of H^+^,K^+^-ATPase monitored by FITC fluorescence.** *A*, dose-dependent inhibition of K^+^-*p*-nitrophenyl phosphatase activity of FITC-modified H^+^,K^+^-ATPase by XFs. Membrane-bound FITC-modified H^+^,K^+^-ATPase preparations were incubated with the indicated concentrations of BeF (*red*), AlF (*green*), or MgF (*yellow*), and their K^+^-*p*-nitrophenyl phosphatase activity was measured (*filled circles* and *lines*). *Open symbols with dotted lines* indicate the dose dependence of XFs on H^+^,K^+^-ATPase activity of the unmodified enzyme (17). The lower table indicates apparent the IC_50_ for each XF used. The values are the mean ± S.D. (*n* = 3) when larger than the symbol. *B* and *C*, EM maps of the cytoplasmic domains of H^+^,K^+^-ATPase in the (SCH)*E2*·BeF (*B*) and that in (SCH)*E2*·MgF states (*C*), superimposed with their respective homology models (color coded as in Fig. 2). EM density responsible for bound ADP (*stick*) is clearly seen in the (SCH)*E2*·BeF state but missing in the (SCH)*E2*·MgF state (*black arrowheads*). The FITC-binding site (Lys-518) at the nucleotide binding pocket is indicated by *spheres* in each map. Schematic representations of each conformational state are shown on the *upper left* (see Fig. 9 for details). *D* and *E*, changes in FITC fluorescence intensities in response to the addition of indicated ligands. *D*, a representative of the time-course experiment of FITC fluorescence intensity. FITC-modified H^+^,K^+^-ATPase membrane fractions were incubated at 37 °C, and their fluorescence intensity changes (Δ*F*, scale for 20% change is shown in the *right*) were monitored. *White* and *black arrowheads* indicate time of the addition of phosphate, its analogs, or SCH28080 (*SCH*) to the sample, respectively. *E*, amount of Δ*F* after the addition of the indicated reagents is shown. *Open* and *closed columns* indicate the absence or presence of SCH28080, respectively, with the indicated phosphate (analogs) added as shown at the *bottom*. Data displayed show the mean ± S.D. from three independent experiments. Expected reaction states are shown in *parentheses*.

The fluorescence probe FITC preferentially forms a covalent bond with the ϵ-amino group of the Lys-518 residue, which is embedded in the conserved Lys-518 in the ATP binding site of the N domain (38). This chemical modification of the Lys residue impairs H^+^,K^+^-ATPase activity (1.7% of residual H^+^,K^+^-ATPase activity compared with that of mock-treated enzyme) due to a loss of ATP-binding ability, suggesting that the FITC probe is located at the nucleotide binding position. The FITC-modified H^+^,K^+^-ATPase, however, can hydrolyze substrates less bulky than ATP, such as acetyl phosphate or *p*-nitrophenyl phosphate (39), showing 76% residual K^+^-*p*-nitrophenyl phosphatase activity compared with that of mock-treated enzyme. The FITC-modified enzyme also has affinities for XFs comparable with those of the unmodified enzyme (Fig. 4*A*). Therefore, the FITC-modified enzyme remains active and undergoes a conformational change in response to substrate binding, allowing us to monitor the conformational changes, especially those occurring at the nucleotide binding site in the N domain, based on the fluorescence intensity (40).

To determine whether the observed rearrangement of the cytoplasmic domains in the H^+^,K^+^-ATPase structure in fact occurred in the solution or was an artifact due to the crystal condition and/or packing, the conformational change in the enzyme in response to XF and SCH28080 binding was evaluated using FITC fluorescence as a probe (Fig. 4). Because the rotational motion of the A domain between (SCH)*E2*·BeF and (SCH)*E2*·MgF (Fig. 3, *A* and *B*) also changes the distance between the A and N domains in each structure, which may affect bound ADP at the nucleotide binding site in the N domain (Fig. 4, *B* and *C*). Bound ADP is likely to be coordinated to both the N domain and the outermost A domain, as seen in the (SCH)*E2*·BeF state in which the distance between the A and N domains is shortest among all the other *E2*P-related structures (Fig. 4*B*). Therefore, changes in the distance between the A and N domains are related to the absence of the EM density responsible for the bound ADP in the (SCH)*E2*·MgF (A domain ∼20° rotated, as shown in Fig. 4*C*) as well as in (Rb^+^)*E2*·AlF states (A domain ∼20° rotated and ∼5° inclined, as shown in Fig. 3, *E* and *F*).

We found that the addition of BeF increased the FITC fluorescence signal (6.9 ± 1.2% increase), whereas that of MgF decreased it (8.3 ± 0.6% decrease). In every experiment the addition of SCH28080 to the respective condition slightly decreased the fluorescence signal (Fig. 4*D*). The fluorescence intensity rapidly increased in response to the addition of BeF (Fig. 4*D*, τ_0.5_ < 1.3 s), consistent with its high affinity binding (17) and the fast inhibition of the ATPase activity of the FITC-free enzyme in the presence of 1 mm BeF (not shown). The addition of Mg^2+^ and P_i_ (MgP_i_) induced a genuine *E2*P formation, which also increased the fluorescence signal (7.7 ± 1.0%), suggesting that BeF acts as a close mimetic of genuine acylphosphate as reported for SERCA (20). The rate of *E2*^32^P formation from radioactive ^32^P_i_ (τ_0.5_ = ∼15 s at 20 °C, from Ref. 44, or 23 s at 0 °C^3^) was close to that of the fluorescence increase occurring after the addition of MgP_i_ (Fig. 4*D*, τ_0.5_ = 19 s at 25 °C), supporting the notion that the observed change in fluorescence intensity was due to *E2*P formation. Although the addition of MgF decreased the signal intensity, the slow response of the fluorescence signal (Fig. 4*D*, τ_0.5_ = 71 s) was consistent with its slow inhibition of ATPase activity (τ_0.5_ = 120 s, from Ref. 17), thus reflecting its binding to the enzyme. Unfortunately, FITC fluorescence measurements are not applicable to the AlF-bound form, because the addition of AlF induces a large amount of nonspecific quenching of the FITC fluorescence intensity, making it unlikely to reflect the conformational change of the enzyme (Fig. 4*D*, *gray trace*), probably due to a direct reaction between AlF and the FITC dye itself (45).

Because FITC fluorescence intensity generally increases in a hydrophobic environment, bound FITC located at the nucleotide binding site might be surrounded by the outermost amino acids of the A domain, just like bound ADP in the (SCH)*E2*·BeF condition (Fig. 4*B*). In contrast, as indicated by the decreased fluorescence intensity in the (SCH)*E2*·MgF condition, bound FITC might be exposed to the bulk solution due to the separated N and A domains in the (SCH)*E2*·MgF state in which bound ADP is disordered (Fig. 4*C*). Bound ADP was also visible in the SCH28080-free *E2*·BeF state (17), the condition in which FITC fluorescence intensity was increased (Fig. 4, *D* and *E*). Therefore, although fluorescence measurements cannot be used for the AlF-bound form, the result is consistent with the appearance of the ADP density in other available XF-bound cryo-EM structures of H^+^,K^+^-ATPase, and thus the observed rearrangements in the cytoplasmic domains are likely occur in the native condition.

##### Transmembrane Helices

**FIGURE 5.**
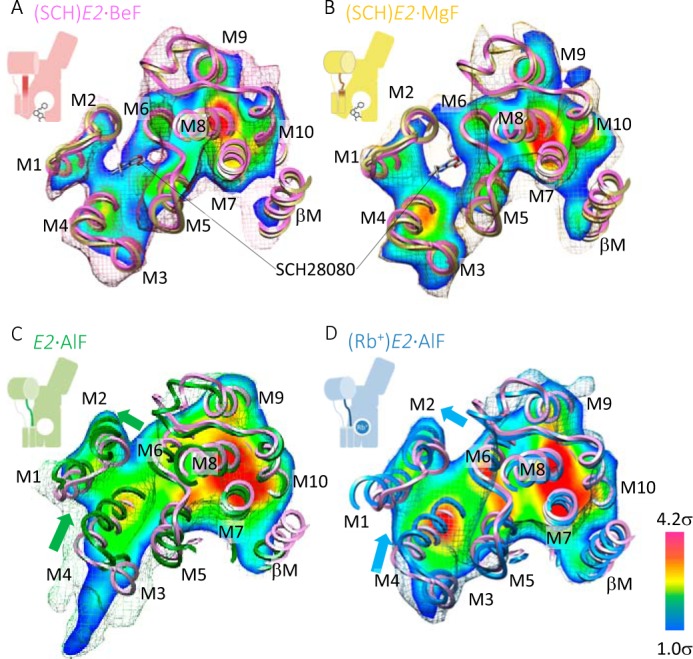
**Comparisons of the TM helices.** *A–D*, cross-sections parallel to the membrane plane for the luminal TM region of the indicated conformational states (*A*, (SCH)*E2*·BeF; *B*, (SCH)*E2*·MgF; *C*, *E2*·AlF; *D*, (Rb^+^)*E2*·AlF) is viewed from the luminal side of the membrane, with their homology models superimposed (*ribbons*). *Mesh* represents the outer surface of the EM maps (1σ). The surface color in the cross-sections indicates the contour level at the indicated plane, gradually changing from *blue* (1 σ) to *red* (4.2 σ) as indicated on the *lower right*. For comparison of the arrangement of TM helices in each state, a homology model for the (SCH)*E2*·BeF state (*magenta ribbon*) is shown in all panels. *Green* and *blue arrows* in *C* and *D* indicate displacement of the TM helices from the (SCH)*E2*·BeF to *E2*·AlF and (Rb^+^)*E2*·AlF states, respectively. Schematic representations of their conformational states are shown on the *upper left of each panel* (see Fig. 9 for details).

Like in the (SCH)*E2*·BeF state, bound SCH28080 imposed a luminal-open conformation of TM helices in the (SCH)*E2*·MgF state (Fig. 5, *A* and *B*). Comparison between the luminal-open conformation of the SCH28080-bound forms and the luminal-closed conformation of *E2*·AlF and (Rb^+^)*E2*·AlF revealed that opening and closing of the luminal helices was accomplished by rearranging the M1-M4 helices (Fig. 5, *C* and *D*). During the transition from an open to closed form of the luminal helices, the luminal half of the M3M4 helices was shifted laterally to close the SCH28080-binding site, which was coupled with the movement of the M1M2 helices (see Fig. 5, *C* and *D*, *green* and *blue arrows*).

##### Affinity and Binding Mode of SCH28080 with Different XFs

**FIGURE 6.**
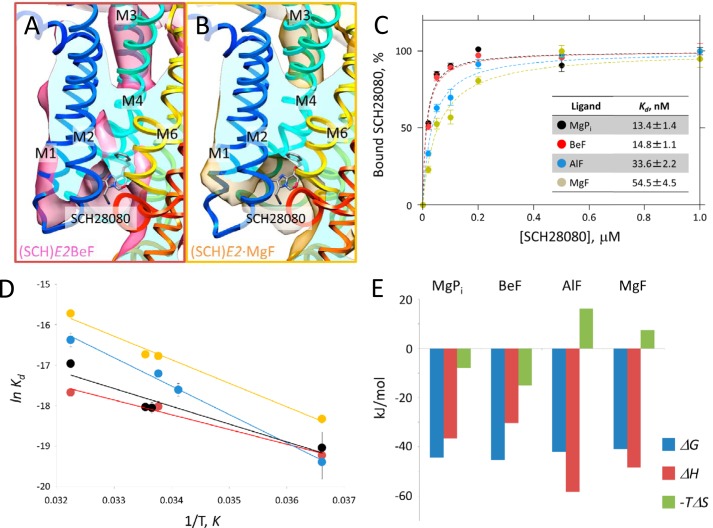
**SCH28080 binding to the H^+^,K^+^-ATPase.** *A* and *B*, close-up view of the SCH28080-binding site at the luminal cavity in the (SCH)*E2*·BeF (*A*) and (SCH)*E2*·MgF (*B*) states. Molecular surface representation of each EM density map (1σ) with superimposed homology models (color gradually changes from M1 (*blue*) to M10 (*red*) as indicated). Cross-sections of the SCH28080-binding site are shown as a *light blue surface*. The figures were viewed from parallel to the membrane plane (cytoplasmic side-up). *C*, dose dependence of SCH28080 binding to the *E2*P and its analog states. Purified H^+^,K^+^-ATPase membrane fractions were incubated in the presence of the indicated ligands followed by the addition of [^3^H]SCH28080. Data shown are the mean ± S.E. (*n* = 3), measured at 25 °C. The *inset table* shows the affinity (*K_d_*) ± S.E. for the SCH28080 binding in each indicated condition. *D*, van't Hoff plot for SCH28080 binding. *K_d_* values were determined directly from three independent binding experiments at each temperature (K). Colors for each symbol are as in *C*. Values are the mean ± S.E. (*n* = 3). *E*, the contribution of enthalpy and entropy to the free energy of SCH28080 binding. Values for enthalpy (Δ*H*, *red columns*) and entropy (Δ*S*, *green columns*) were obtained from the van't Hoff relationship (Equation 1) for each indicated condition, as described under “Experimental Procedures.” These values were then used to calculate the corresponding free energies (Δ*G*, *blue columns*, calculated as 25 °C) using Equation 2.

Despite the identical luminal-open arrangement of TM helices in the (SCH)*E2*·BeF and (SCH)*E2*·MgF states, however, most of the SCH28080 moiety was invisible in the density map of (SCH)*E2*·MgF (Fig. 6*B*), which is in marked contrast to that of (SCH)*E2*·BeF (Fig. 6*A*). Because SCH28080 was required for the two-dimensional crystallization in the (SCH)*E2*·MgF condition as well as the different arrangement of the TM helices in (SCH)*E2*·AlF from its absent *E2*·AlF state (Fig. 5*C*), the observed poor density at the SCH28080-binding site was likely due to the disorder of this antagonist at the luminal cavity. Such a difference around the presumed SCH28080 density in the (SCH)*E2*·BeF and the (SCH)*E2*·MgF states prompted us to investigate the SCH28080 binding affinity to H^+^,K^+^-ATPase bound to the various XFs (Fig. 6*C*). Using ^3^H-labeled SCH28080, the effects of XFs on the SCH28080 binding affinity were examined (41). BeF-bound H^+^,K^+^-ATPase had a high affinity for the SCH28080 binding (*K_d_* = 14.8 ± 1.1 nm), which was as high as that for the genuine *E2*P formed from MgP_i_ (*K_d_* = 13.4 ± 1.4 nm) at 25 °C. In contrast, AlF- or MgF-bound H^+^,K^+^-ATPase had 3∼4 times lower affinity for SCH28080 (*K_d_* = 33.6 ± 2.2 nm, *K_d_* = 54 ± 4.5 nm) than BeF-bound H^+^,K^+^-ATPase, consistent with the observed poor density of SCH28080 at the luminal cavity in the (SCH)*E2*·MgF (Fig. 6*B*) and (SCH)*E2*·AlF (not shown) states.

##### A-M2 Linker That Connects Cytoplasmic Domains and TM Helices

**FIGURE 7.**
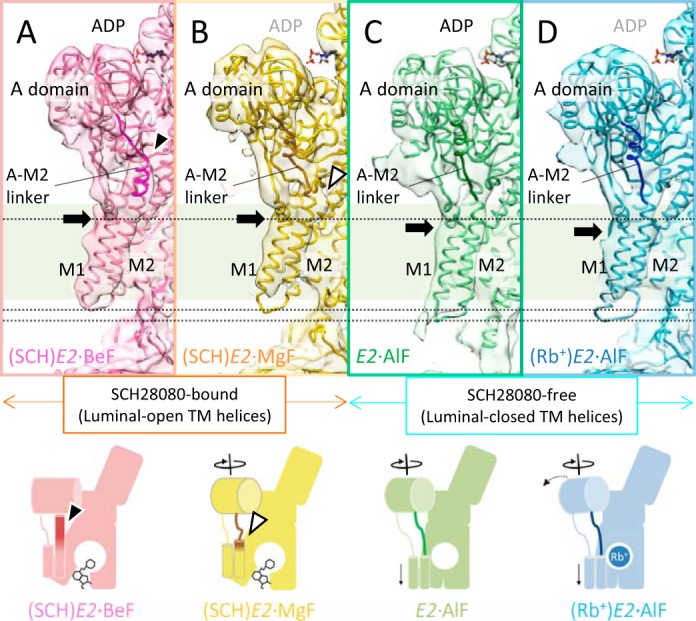
**Conformational changes in the A-M2 linker.** *A–D*, close-up view of the A-M2 linker (Glu-160–Gln-176, indicated by *dark colors* in homology models) in the reaction states of (SCH)*E2*·BeF (*A*), (SCH)*E2*·MgF (*B*), *E2*·AlF (*C*), and (Rb^+^)*E2*·AlF (*D*). Each molecular surface and ribbon model represents the EM density map (1σ) and homology model of the corresponding states, respectively. *Black arrows* show the position of the characteristic kink region found in the cytoplasmic portion of M1, which indicates the vertical shift of the M1M2 helices between the SCH28080-bound states (*A* and *B*) and SCH28080-free states (*C* and *D*). *Dotted lines* also serve as references for the vertical shift of the M1M2 helices. Although the EM density at the luminal portion of M1M2 is missing due to flexibility-induced disorder, the location of M1M2 is significantly different between the SCH28080-bound (*A* and *B*) and its free (*C* and *D*) conditions. The *black arrowhead* indicates the upright α-helical conformation of the A-M2 linker in the (SCH)*E2*·BeF state (*A*). The *white arrowhead* indicates the protruding EM density observed in the (SCH)*E2*·MgF state (*B*). The schematic representations of their conformational states are shown on the lower panel (see Fig. 9 for details).

**FIGURE 8.**
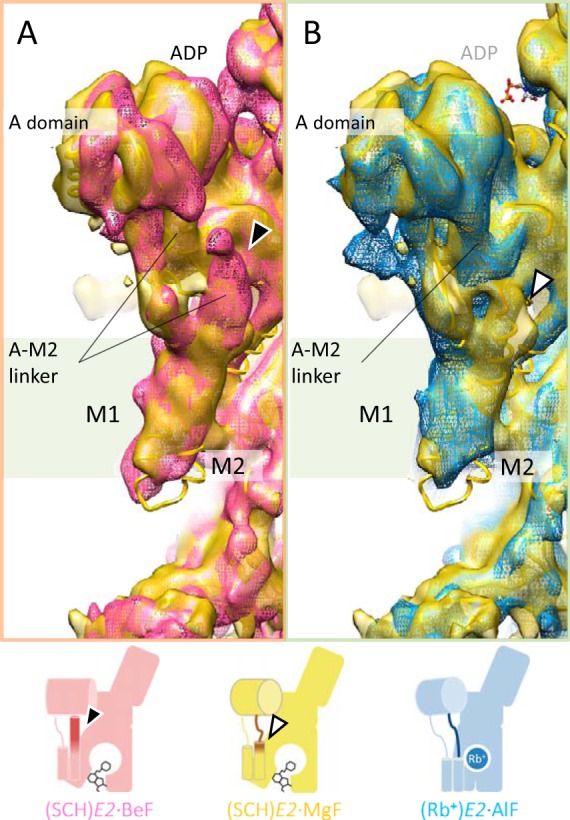
**Comparison of the molecular conformation between the (SCH)*E2*·MgF state and the (SCH)*E2*·BeF (*A*) or (Rb^+^)*E2*·AlF states (*B*).** Only (SCH)*E2*·MgF is shown as a surface representation with a homology model superimposed (*yellow*), whereas EM maps of other states are shown in *mesh representations*. The *black arrowhead* indicates the upright α-helical conformation of the A-M2 linker in the (SCH)*E2*·BeF state (*A*, *red mesh*). The *white arrowhead* indicates the protruding EM density observed in the (SCH)*E2*·MgF state (*B*), which reflects the vertical shift of M2 compared with that in the (Rb^+^)*E2*·AlF state (*blue mesh*). Schematic representations of the compared states are shown in the *lower panel* (see Fig. 9 for details).

Rotation of the A domain was in turn transmitted to the TM region, which was mediated by the connecting linker between them (Fig. 7). The middle of the A-M2 linker, indicated as *dark-colored tubes* in Fig. 7, *A–D*, assumed an unwound loop structure in the (SCH)*E2*·MgF state (Fig. 7*B*) almost identical to that in the *E2*·AlF and (Rb^+^)*E2*·AlF states (Fig. 7, *C* and *D*, respectively), but significantly different from that in the (SCH)*E2*·BeF state (Fig. 7*A*). The conformation of the juxta-membranous portion of the A-M2 linker and M2 helix in (SCH)*E2*·MgF, however, was largely different from those in *E2*·AlF and (Rb^+^)*E2*·AlF, but is similar to that in (SCH)*E2*·BeF (Fig. 7*A*, Fig. 8). These differences were related to the conformational rearrangement of the A domain and TM helices (Figs. 3, 5). In particular, the luminal-open TM arrangement induced by SCH28080 binding was accompanied by a lateral shift of the M3M4 helices (Fig. 5) and at the same time a vertical shift of M1M2 helices toward the luminal side, as seen in the different vertical orientations of the kink region of the A-M1 linkers (Fig. 7, *A–D*, *black arrows*) and the characteristic protrusion of the EM density at the end of M2 in the (SCH)*E2*·MgF state (Figs. 7*B* and 8*B*, *white arrowhead*). In the (SCH)*E2*·MgF state, the cytoplasmic portion assumed a typical “luminal-closed” form (∼20° rotated A domain and unwound loop structure of the A-M2 linker), which might subsequently induce the luminal gate closure in the absence of SCH28080 as seen in the *E2*·AlF structure (Fig. 5*C*). The M1M2 helices, however, could not move due to the luminal-open conformation of the TM helices fixed by bound SCH28080 at the luminal cavity. As a result, the (SCH)*E2*·MgF structure represents a hybrid conformation, with the relative orientation of the cytoplasmic domains similar to that in a typical luminal-closed type conformation (Figs. 3 and 7) and the TM region assuming a luminal-open conformation (Fig. 5).

## DISCUSSION

In this paper we describe two identical cryo-EM structures of H^+^,K^+^-ATPase in the (SCH)*E2*·AlF and (SCH)*E2*·MgF states. Including these newly determined structures, all *E2*P-related structures of H^+^,K^+^-ATPase were crystallized in the presence of phosphate analogs with or without the synthetic antagonist SCH28080. The phosphate analogs used in these studies are well characterized for SERCA (20, 21) and are also applicable for H^+^,K^+^-ATPase (17, 19); thus, they likely act as close mimetics of reaction substeps of the phosphorylated form of the enzyme. The K^+^-competitive antagonist, SCH28080, binds to the luminal cavity, which connects with the cation binding site in the middle of the TM helices (18). The arrangement of the TM helices in the SCH28080-bound structures of H^+^,K^+^-ATPase is surprisingly similar to that in the *E2*·BeF structure of SERCA in which the cation binding site is exposed to the luminal solution (21, 22). Therefore, it is most likely that the luminal-open TM arrangement imposed by SCH28080 mimics the situation in which the luminal gate is opened to exchange H^+^/K^+^ to the luminal solution. As protein crystal structures with bound artificial antagonists or inhibitors are reported to reveal a snapshot of the protein at work, it is not surprising that an artificial antagonist utilizes the binding site exposed when the protein is in a certain conformation (46–48). We, therefore, conclude that the molecular conformations induced by XFs and their combination with SCH28080 might occur in the native enzyme.

**FIGURE 9.**
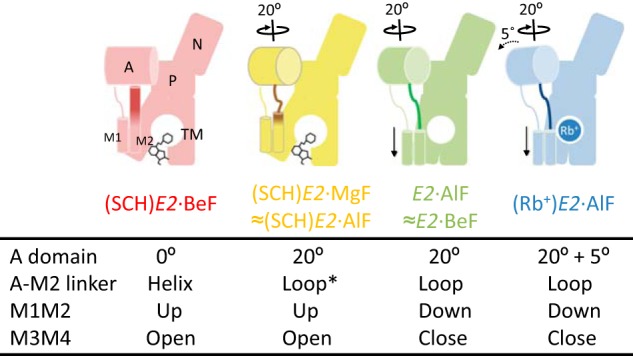
**Schematic drawing of *E2*P analog conformations of H^+^,K^+^-ATPase induced by XF and SCH28080.** Molecular conformation of (SCH)*E2*·BeF (*red*), (SCH)*E2*·MgF (*yellow*), *E2*·AlF (*green*), and (Rb^+^)*E2*·AlF (*blue*) are shown as simplified schematics. Three cytoplasmic domains and TM domains are indicated in the figure. Bound SCH28080 and Rb^+^ are shown as *black sticks* and *blue circle*, respectively, and the A-M2 linker is highlighted by a *dark color* in each reaction state. *Two thin cylinders* indicate M1M2 helices. *Arrows* indicate the conformational difference when each state is compared with the (SCH)*E2*·BeF state. The *lower table* indicates conformational features of key components for each reaction state. *A domain*, the azimuthal positions of the A domain in each state; that of (SCH)*E2*·BeF is set as 0° (Fig. 3). *A-M2 linker*, conformation of the A-M2 linker (Fig. 7). The *asterisk* indicates loosened A-M2 linker in the (SCH)*E2*·MgF state (Fig. 7*B*). *M1M2*, vertical location of the M1M2 helices. *Up* and *Down* represent the vertical location of the helices on the relatively cytoplasmic side and luminal side, respectively, in each state (Fig. 7). *M4M5*, arrangement of the luminal half of the M3M4 helices (Fig. 5).

As discussed below, H^+^,K^+^-ATPase may inherently prefer the luminal-closed conformation because, even when using BeF, which drives SERCA into the luminal-open conformation, H^+^,K^+^-ATPase closes its luminal gate (17). This makes structural investigation of luminal-gating mechanism of H^+^,K^+^-ATPase particularly difficult if using only XFs as a conformational modulator. Using SCH28080 to impose the luminal-open conformation allows us to address the mechanistic rationale for the luminal gating of H^+^,K^+^-ATPase. Systematic comparison of the molecular conformations reveals several key components and how they regulate the luminal gating of H^+^,K^+^-ATPase (Fig. 9).

To our surprise the newly determined (SCH)*E2*·MgF structure likely represents a chimeric conformation between the luminal-open (SCH)*E2*·BeF (18) and luminal-closed (Rb^+^)*E2*·AlF (19) states (Fig. 9), a conformation that has until now been inaccessible for SERCA and other P-type ATPases. For ATP-driven P-type ATPases, however, a conformational change of the ATP-hydrolyzing cytoplasmic domains must in principle be tightly coupled to that in the cation-transporting TM helices. This principle is supported by several crystal structures of SERCA in *E2*P analog states in which the luminal gate closure is coupled with a 20° rotational motion of the A domain via a connecting unwound structure, the A-M2 linker (16, 21). In this respect, the molecular conformation in the observed hybrid structure of the H^+^,K^+^-ATPase (SCH)*E2*·MgF state is uncoupled in terms of the conformational relationship between the cytoplasmic domains and the TM region. Comparison of the hybrid structure with the luminal-open and luminal-closed structures revealed that two contradicting motions, a 20° rotational motion of the A domain on the cytoplasmic side that drives the luminal-closed TM arrangement (Fig. 3) and the luminal-open arrangement in the TM helices fixed by the SCH28080-binding (Fig. 5), are both transmitted to the connecting A-M2 linker (Fig. 7). As a result, this linker region (more specifically, its boundary with the M2 helix; Fig. 8) is constrained in the hybrid conformation of the (SCH)*E2*·MgF state (Fig. 9). Hence, the A-M2 linker acts as a switching element between two portions of the enzyme, thereby mediating an important role for the correct coupling of the ATP hydrolysis and luminal gating in physiologic situations.

Systematic comparison of molecular conformations in SCH28080-bound *E2*P-related structures of H^+^,K^+^-ATPase revealed that the azimuthal position of the A domain in each XF-bound structure reflects the coordination geometries of each bound XF in SCH28080-bound conditions (Fig. 9), as observed in the SERCA structures (21, 22). Closure of the luminal gate is accompanied by a lateral shift of the M3M4 helices (Fig. 5, *C* and *D*), and a vertical shift of the M1M2 helices toward the luminal side (Fig. 7, *A–D*). In particular, the latter shift induces a conformational change of the A-M2 linker and subsequently forces the A domain into a 20° rotated position like in the *E2*·AlF and (Rb^+^)*E2*·AlF states (Fig. 3). When the bound SCH28080 imposes a luminal-open form on the TM helices, the A-M2 linker might be loosened (Figs. 7 and 8) because the M1M2 helices must move vertically to a more cytoplasmic side than in the luminal-closed form. Therefore, in the SCH28080-bound forms, the A domain can freely rotate in response to the coordination geometry of each bound XF. This might be why the azimuthal position of the A domain in each different XF-bound structure reflects the coordination geometry of the respective XF in the presence of SCH28080. In the BeF-bound form and probably in the genuine phosphate-bound form as well, the A domain remains at a 0° rotated position (Fig. 3*A*), which permits tight coordination of the aspartyl phosphate (or its analog, Asp-BeF) by the TGES loop when SCH28080 is bound to the luminal cavity, thereby allowing the loosened A-M2 linker to assume an α-helical structure extended from the M2 helix (Figs. 7*A* and 8*A*). In contrast, in the case of the AlF- or MgF-bound forms, even if SCH28080 is bound, the A domain remains in the 20° rotated position (Fig. 3*A*) because these XFs sterically restrain the A domain in the 0° rotational position, in which the expected position of the TGES loop seems too close to coordinate with them appropriately.

Notably, H^+^,K^+^-ATPase tends to accumulate in the luminal-closed conformation (Fig. 5*C*) in which the A domain is in a 20° rotated position relative to the (SCH)*E2*·BeF structure (Fig. 3, *C* and *D*). In contrast to SERCA, even if BeF is used as the closest analog of aspartyl phosphate in the absence of SCH28080 (17), H^+^,K^+^-ATPase adopts an identical structure in the luminal-closed *E2*·AlF state. These observations corroborate the notion that the TM helices of H^+^,K^+^-ATPase prefer the luminal-closed conformation to reduce the risk of H^+^ reverse flow from the highly acidic gastric lumen (13, 14). Because of tight coupling between the TM helices and cytoplasmic domains, this preference of the TM helices may be strong enough to influence the arrangement of cytoplasmic domains, especially the A domain, via the A-M2 linker in the case of H^+^,K^+^-ATPase. Therefore, we speculate that the molecular conformation of H^+^,K^+^-ATPase in a genuine *E2*P state is mainly the luminal-closed conformation like in the *E2*·AlF and *E2*·BeF states to prevent the reverse reaction with the isolation of the proton binding site from the highly acidic environment (13). Subsequent K^+^ binding, however, requires the formation of a physical pathway from the luminal bulk solution to the cation binding site. The hybrid structure of (SCH)*E2*·MgF described in the present paper suggests the entity of such a luminal-open conformation of the TM helices without displacement of the A domain, even for a short time. Therefore, for the subsequent dephosphorylation of *E2*P in the transport cycle, K^+^ may enter when the thermal fluctuation of the TM helices induces a conformation such as (SCH)*E2*·MgF to allow for physical access of K^+^ to the cation binding site from the luminal solution.

To determine the thermodynamic parameters for SCH28080 binding, we evaluated the effect of the temperature on the SCH28080 binding to *E2*P or the XF-inhibited *E2*P analog states. Overall changes in enthalpy (Δ*H*) and entropy (Δ*S*) accompanied by SCH28080 binding were obtained from the van't Hoff relationship (Fig. 6, *D* and *E*). SCH28080 binding studies revealed that the *E2*P or analogous states of *E2*P fall into two groups. *E2*P and *E2*·BeF show high affinity with both Δ*H* and −*T*Δ*S* favorable for binding free energy. In contrast, *E2*·AlF and *E2*·MgF show lower affinity with an unfavorable −*T*Δ*S* term. Because an unfavorable entropy term usually reflects a large degree of flexibility, this could explain the observed poor density for bound SCH28080 in the (SCH)*E2*·AlF and (SCH)*E2*·MgF states (Fig. 6*B*). The SCH28080 binding pocket structures (*i.e.* arrangements of TM helices) in all SCH28080-bound *E2*P states were indistinguishable from their EM maps (Fig. 5, *A* and *B*). We speculated that the different conformations of the A domain contribute to the different affinities and stabilities of the molecular conformation in each state. In the (SCH)*E2*·AlF (not shown) and (SCH)*E2*·MgF states, the A domain locates at a 20°-rotated position, which drives luminal gate closure by the unwinding of the A-M2 linker and subsequent downward movement of the M1M2 helices when SCH28080 is dissociated. Thus, although the TM helices in (SCH)*E2*·MgF are forced into the luminal-open conformation by bound SCH28080, the TM helices may always face conflicting pressure of the gate closure from the cytoplasmic A domain. Such a conformational dynamics may be related to the lower affinity of SCH28080 in the (SCH)*E2*·MgF state. Because the enthalpy-driven binding mode often involves conformational changes of the protein, the determined thermodynamic parameters may reflect conformational changes of the enzyme upon SCH28080 binding rather than its simple binding without conformational changes, like a lock and key. Hence, the different binding parameters observed in the two groups (*E2*P and *E2*·BeF *versus E2*·AlF and *E2*·MgF) suggest that SCH28080 binding induces different conformations.

Here we highlighted the important contribution of the A-M2 linker in gating the ion pathway on the luminal side of H^+^,K^+^-ATPase by determining a hybrid conformation and comparing it with luminal-open and luminal-closed conformations. The accumulation of examples of the conformational changes in SERCA and in other P-type ATPases allows for their comparison (49–52). A recently reported structure of the high affinity Na^+^,K^+^-ATPase-ouabain complex with bound genuine phosphate (36) shows close similarity to the H^+^,K^+^-ATPase (SCH)*E2*·BeF structure (18), suggesting that these two closely related ATPases have a similar mechanism for high affinity inhibitor binding as well as for the conformational change accompanied by luminal gate opening, which was well predicted from the structure of MgF-bound Na^+^,K^+^-ATPase with bound K^+^ and ouabain (33). Structural comparison of the *E2*·BeF and *E2*·AlF states of CopA, a Cu^2+^-transporting PIB-type ATPase, also shows a very similar conformational rearrangement of the A domain and A-M2 linker induced by the binding of these two respective XFs in H^+^,K^+^-ATPase, although a unique luminal transport pathway has been proposed (52). Such surprising similarities over the different subfamilies of P-type ATPases suggest that the basic mechanism for luminal gating coupled with conformational rearrangement of the cytoplasmic domains is likely conserved for P-type ATPase in general.

## Footnotes

3 K. Abe, unpublished data.

## Footnotes

2 The abbreviations used are:

SERCA: sarcoplasmic reticulum Ca^2+^-ATPase
TM: transmembrane
BeF: beryllium fluoride (BeF_3_^−^)
AlF: aluminum fluoride (AlF_4_^−^)
MgF: magnesium fluoride (MgF_4_^2−^)
XF: fluorinated phosphate analog.
